# Tuberculosis infection and stillbirth in Ethiopia—A prospective cohort study

**DOI:** 10.1371/journal.pone.0261972

**Published:** 2022-04-11

**Authors:** John Walles, Laura García Otero, Fregenet Tesfaye, Asmamaw Abera, Marianne Jansson, Taye Tolera Balcha, Erik Sturegård, Niclas Winqvist, Stefan R. Hansson, Per Björkman

**Affiliations:** 1 Clinical Infection Medicine, Department of Translational Medicine, Lund University, Malmö, Sweden; 2 Department of Infectious Diseases, Central Hospital, Kristianstad, Sweden; 3 Fetal Medicine Research Center, BCNatal—Barcelona Center for Maternal-Fetal and Neonatal Medicine, University of Barcelona, Barcelona, Spain; 4 Institut Clinic de Ginecologia, Obstetricia i Neonatologia (ICGON), Institut d’Investigacions Biomèdiques August Pi i Sunyer (IDIBAPS), University of Barcelona, Barcelona, Spain; 5 Universitat de Barcelona, and Centre for Biomedical Research on Rare Diseases (CIBER-ER), University of Barcelona, Barcelona, Spain; 6 Armauer Hansen Research Institute, Addis Ababa, Ethiopia; 7 Ethiopia Institute of Water Resources, Addis Ababa University, Addis Ababa, Ethiopia; 8 Medical Microbiology, Department of Laboratory Medicine, Lund University, Lund, Sweden; 9 Clinical Microbiology, Division of Laboratory Medicine, Lund, Sweden; 10 Skåne Regional Office for Infectious Disease Control and Prevention, Malmö, Sweden; 11 Division of Obstetrics and Gynecology, Department of Clinical Sciences Lund, Lund University, Lund, Sweden; 12 Skåne University Hospital, Lund, Sweden; 13 Department of Infectious Diseases, Skåne University Hospital, Malmö, Sweden; University of Washington, UNITED STATES

## Abstract

**Background:**

Tuberculosis is among the leading causes of death among infectious diseases. Regions with a high incidence of tuberculosis, such as sub-Saharan Africa, are disproportionately burdened by stillbirth and other pregnancy complications. Active tuberculosis increases the risk of pregnancy complications, but the association between latent tuberculosis infection (LTBI) and pregnancy outcomes is unknown. We explored the effect of latent tuberculosis infection on the risk of stillbirth in women attending antenatal care clinics in Ethiopia, a country with >170 000 annual cases of active tuberculosis.

**Method:**

Pregnant women were enrolled from antenatal care at three health facilities in Adama, Ethiopia, during 2015–2018, with assessment for previous and current active tuberculosis and testing for LTBI using QuantiFERON-TB-GOLD-PLUS. Proportions of stillbirth (≥ 20 weeks of gestation) and neonatal death (< 29 days of birth) were compared with respect to categories of maternal tuberculosis infection (tuberculosis-uninfected, LTBI, previous-, and current active tuberculosis). Multivariable logistic regression was performed for stillbirth.

**Results:**

Among 1463 participants enrolled, the median age was 25 years, 10.2% were HIV-positive, 34.6% were primigravidae, and the median gestational age at inclusion was 18 weeks. Four (0.3%) were diagnosed with active tuberculosis during pregnancy, 68 (4.6%) reported previous treatment for active tuberculosis, 470 (32.1%) had LTBI, and 921 (63.0%) were tuberculosis-uninfected. Stillbirth was more frequent in participants with LTBI compared to tuberculosis-uninfected participants, although not reaching statistical significance (19/470, 4.0% vs 25/921, 2.7%, adjusted [for age, gravidity and HIV serostatus] odds ratio 1.38, 95% confidence interval 0.73–2.57, p = 0.30). Rates of neonatal death (5/470, 1.1% vs 10/921, 1.1%) were similar between these categories.

**Conclusion:**

Latent tuberculosis infection was not significantly associated with stillbirth or neonatal death in this cohort. Studies based on larger cohorts and with details on causes of stillbirth, as well as other pregnancy outcomes, are needed to further investigate this issue.

## Introduction

Sub-Saharan Africa carries a disproportionately high burden of maternal mortality, stillbirth, and neonatal death [1–3]. Recent multinational studies have reported that infectious diseases may contribute to 25–60% of stillbirths and half of neonatal deaths in this world region [4,5]. Pregnant women have increased susceptibility to infectious diseases due to transient physiological immune modulations [6]. In addition, several infectious diseases, such as HIV and malaria, can contribute to adverse pregnancy outcomes [7].

Apart from high rates of maternal mortality, stillbirth and neonatal deaths, sub-Saharan Africa also has a high incidence of TB [8]. TB is among the leading infectious causes of morbidity and mortality, globally accounting for more than 1.5 million deaths in 2020 (8). The role of tuberculosis (TB) in the context of pregnancy is debated [9,10]. Compared to other world regions, women living in sub-Saharan Africa have a relatively increased risk of active TB; this has been associated with higher HIV prevalence among women [11]. In addition, it is possible that pregnancy could be involved in this phenomenon, as two register-based studies in Sweden and the United Kingdom have indicated increased incidence of active TB in connection to pregnancy [12,13]. Active TB occurs in around 200 000 pregnant women annually [14] and is a major cause of maternal mortality, contributing to nearly one fourth of non-obstetric maternal deaths in autopsy studies from sub-Saharan Africa [15–18]. Furthermore, active TB has been associated with adverse pregnancy outcomes, including intrauterine growth restriction, stillbirth and perinatal mortality and morbidity [9,10,19–21]. Whether latent TB infection (LTBI), a common condition that is estimated to occur in > 25% of the world population [22], has an impact on pregnancy outcomes has not previously been investigated.

The mechanisms leading to TB-related pregnancy complications are not fully elucidated. Possibly, systemic inflammation, which accompanies active TB and is implicated in the pathogenesis of various pregnancy complications [7], may be involved in these phenomena. For the current study, we hypothesized that LTBI may also be associated with adverse pregnancy outcomes.

In this study, we aimed to assess the effect of LTBI on stillbirth and neonatal death in women screened for active and latent TB infection during pregnancy in Ethiopia.

## Methods

### Study setting and procedure

This study was based on a prospective cohort of women attending antenatal care (ANC) in Adama, Ethiopia, during 2015–2018, with follow-up until 4 years after delivery [23] (ClinicalTrials.gov identifier: NCT03305991). The health facilities (Adama Hospital Medical College, and Adama and Geda Health Centres) provide TB and HIV care as well as antenatal and obstetric care, and are localized in urban Adama, a city with approximately 300 000 inhabitants in central Ethiopia. Pregnant women were eligible for inclusion at the first ANC visit for their current pregnancy after providing informed consent. At inclusion, participants were interviewed on socioeconomic and demographic characteristics, medical and obstetric history, and details of the current pregnancy. Physical examination was performed. Gestational age at inclusion was determined based on record of last menstrual period, fundal height and/or ultrasound according to clinical routines. Venous blood for LTBI testing was obtained, using QuantiFERON TB GOLD PLUS (QFT) [24]. HIV testing was performed according to Ethiopian guidelines [25]. Participants showing symptoms or signs suggestive of active TB, as well as all HIV-positive participants (irrespective of clinical manifestations), were asked to provide two morning sputum samples for microbiological analysis of *Mycobacterium tuberculosis*, using smear microscopy, GeneXpert MTB/RIF and liquid culture. Ethiopian guidelines currently recommend symptom-based screening for active TB during routine antenatal care [26]. Diagnosis of active TB could also be based on clinical criteria according to national guidelines [26]. Similar investigations were conducted for study participants presenting symptoms suggestive of active TB during follow-up. Up to 3 follow-up visits were scheduled during pregnancy, as well as 6 weeks after delivery, for assessment of pregnancy outcome. Participants not returning for study visits were traced by phone; in case they had moved out of the uptake area, data on stillbirth and neonatal death were collected through phone interviews.

LTBI was defined as QFT ≥0.35 IU/ml in both or either antigen preparation in the assay [27], in the absence of active TB and/or previous history of active TB. Study participants with QFT <0.35 IU/ml in both antigen preparations and without active TB nor previous history of active TB were classified as TB-uninfected. Spontaneous abortion was defined as intrauterine demise before 20 weeks of gestation, and stillbirth was defined as death between 20 weeks of gestation and delivery, or on the day of delivery [28]. Neonatal death was defined as death within the first 28 days after birth, excluding reasons unrelated to pregnancy [29].

Participants without QFT results, and without past or current active TB, could not be categorized with regard to TB infection status, and were therefore excluded, as were those reporting induced termination of pregnancy, those with twin pregnancies, and those who did not return for a physical study visit after delivery to ascertain pregnancy outcome. However, study participants who reported foetal or neonatal death at phone contact after delivery (despite not returning for the scheduled post-delivery study visit) were included. Study participants with repeated pregnancies during follow-up were retained in the cohort, but only their ongoing pregnancies at inclusion were considered for the current study.

### Statistical analysis

The primary outcome was stillbirth. Neonatal death, caesarean section, and hospitalization ≥24 hours in connection to delivery were considered as secondary outcomes. Rates of the separate outcomes were compared with respect to TB infection category in multivariable logistic regression (for outcomes with crude associations with TB category reaching p<0.20), with adjustment for maternal age (because of its association with LTBI status [23], gravidity and HIV serostatus (because of its effect on pregnancy complications) [22]. Maternal education, marital status, and socio-economic characteristics (number of rooms in residence, availability of electricity and latrine in the household) were included in separate sensitivity analyses for each outcome, to control for residual confounding. Cases were excluded from analyses if data was missing on one or more variables included in the respective model. Statistical analysis was performed using R, version 5.3.1 [30].

### Ethical considerations

The study was approved by the ethical review committees of Lund University, Sweden and the Ministry of Science and Technology, Addis Ababa, Ethiopia. Written informed consent was obtained from all study participants prior to enrolment in the study, on behalf of themselves and their offspring. Study participants were screened for active TB based on symptoms and clinical signs at all study visits, and treatment for active TB was provided at the respective health facility. Prophylactic treatment for LTBI is recommended only for young children with household TB exposure, and people living with HIV [26], and testing for LTBI is currently not recommended in any situation. Since neither testing nor treatment for LTBI is recommended in pregnant women in the Ethiopian TB Program [26], QFT results were not communicated to participants or healthcare providers.

## Results

### Participant characteristics

**Fig 1:**
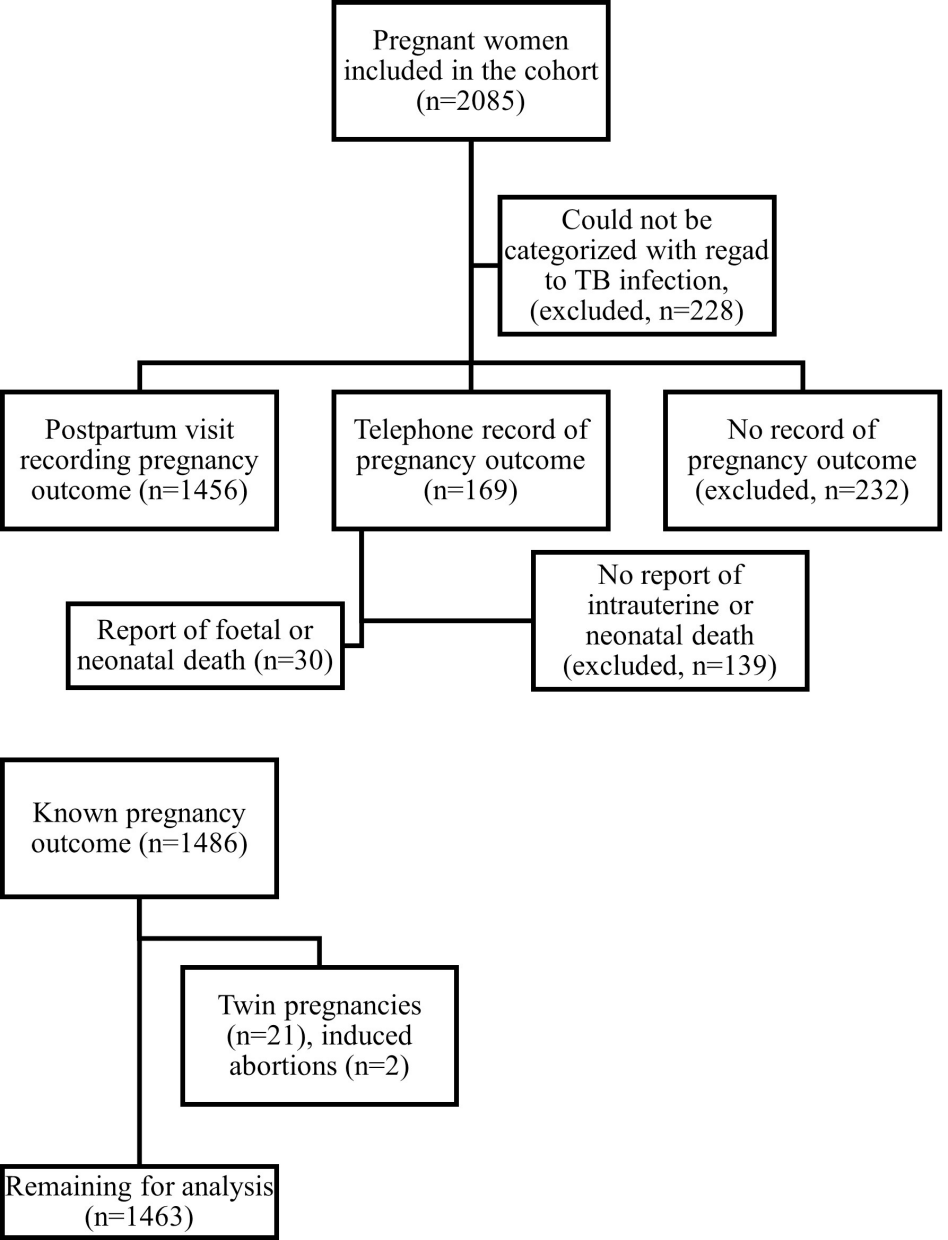
Flowchart of exclusion.

Among 2085 pregnant women included in the cohort, 228 could not be classified with regard to LTBI (QFT results missing, Fig 1). Following the time of scheduled delivery, 1456 had a physical study visit, 169 were reached by telephone, and 232 could not be contacted after delivery. Among those reached by telephone, 30 reported either intrauterine or neonatal death; these were retained in the study together with those with a physical visit after delivery. Among these 1486 study participants, 21 were excluded because of twin pregnancy, and two because of medically induced termination of pregnancy (one because of anencephaly), leaving 1463 for analysis.

Study participants that could not be classified regarding TB infection were excluded, their characteristics were similar to those remaining in the study, as previously reported [23]. Study participants that did not return for a physical visit could still be retained if they reported spontaneous abortion, stillbirth or neonatal death on telephone contact. Participants reporting medically induced termination of pregnancy as well as twin pregnancies were excluded.

Compared to participants remaining for analysis, the 371 participants excluded due to lack of postpartum study visit were younger (24.1 vs 25.2 years, p<0.001), more frequently primigravidae (45.8% *vs* 34.5%, p<0.001), and were less likely to be HIV-positive (4.0% *vs* 10.2% p<0.001) (Table 1). The distribution with regard to TB infection category was similar between included and excluded study participants (TB uninfected 65.8% vs 62.9%) (Table 1).

**Table 1:** Study participant characteristics and pregnancy outcomes by TB infection status.

Characteristics	TB-uninfected^1^ N = 921		LTBI^2^ N = 470		Previous active TB^3^ N = 68		Active TB diagnosed during pregnancy^4^ (n = 4)		Excluded^5^ N = 371	
**DEMOGRAPHIC**	N	%	N	%	N	%	N	%	N	%
**Age (years)**										
**Median, IQR**	25	22–27	26	23–28	26	23–30	24	22–26	24	20–27
**<21**	183	19.9	53	11.3	9	13.2	0	0	102	27.5
**21–25**	400	43.4	168	35.7	20	29.4	3	75.0	140	37.7
**26–30**	278	30.2	192	40.9	29	42.6	1	25.0	106	28.6
**>30**	60	6.5	56	11.9	10	14.7	0	0	21	5.7
**Unknown**	0	0	1	0.2	0	0	0	0	2	0.5
**Marital status**										
**Single**	27	2.9	11	2.3	4	5.9	0	0	11	3.0
**Married**	882	95.8	456	97.0	64	94.1	4	100.0	352	94.9
**Divorced**	9	1	2	0.4	0	0	0	0	4	1.1
**Widow**	2	0.2	0	0	0	0	0	0	1	0.3
**Unknown**	1	0.1	1	0.2	0	0	0	0	3	0.8
**Education**										
**Illiterate**	95	10.3	65	13.8	17	25.0	1	25.0	50	13.5
**< 6 grades**	201	21.8	84	17.9	6	8.8	0	0	75	20.2
**6–12 grades**	536	58.2	267	56.8	34	50.0	1	25.0	200	53.9
**Higher education**	89	9.7	53	11.3	11	16.2	2	50.0	44	11.9
**Unknown**	0	0	1	0.2	0	0	0	0	2	0.5
**Rooms in residence**										
**1**	511	55.5	232	49.4	31	45.6	3	75.0	222	59.8
**≥2**	407	44.2	235	50.0	37	54.4	1	25.0	145	39.1
**Unknown**	3	0.3	3	0.6	0	0	0	0	4	1.1
**Electricity in residence**										
**No**	38	4.1	20	4.3	5	7.4	0	0	11	3.0
**Yes**	878	95.3	445	94.7	62	91.2	4	100.0	357	96.2
**Unknown**	5	0.5	5	1.1	1	1.5	0	0	4	1.1
**Latrine in residence**										
**No**	50	5.4	36	7.7	5	7.4	0	0	15	4.0
**Yes**	868	94.2	430	91.5	63	92.6	4	100	354	95.4
**Unknown**	3	0.3	4	0.9	0	0	0	0	2	0.5
**OBSTETRIC**										
**First pregnancy**										
**No**	565	61.3	336	71.5	52	76.5	1	25.0	198	53.4
**Yes**	352	38.2	134	28.5	16	23.5	3	75.0	170	45.8
**Unknown**	4	0.4	0	0	0	0	0	0	3	0.8
**Gestational age at inclusion (weeks)**										
**Median (IQR)**	18	14–20	18	14–22	18	13–20	14	11–14		
**<14**	143	15.5	84	17.9	18	26.5	2	50.0	57	15.4
**14–27**	555	60.3	266	56.6	40	58.8	2	50.0	208	56.1
**>27**	31	3.4	17	3.6	3	4.4	0	0	29	7.8
**Unknown**	192	20.8	103	21.9	7	10.3	0	0	77	20.8
**HIV-RELATED**										
**HIV status**										
**HIV negative**	845	91.7	426	90.3	40	58.8	2	50.0	356	96.0
**HIV positive**	76	8.3	44	9.4	28	41.2	2	50.0	15	4.0
HIV diagnosis^6^										
**During current pregnancy**	10	13.2	6	13.6	1	3.6	0	0	2	13.3
**Before current pregnancy**	56	73.7	32	72.7	25	89.3	2	100	10	66.7
**Unknown**	10	13.2	6	13.6	2	7.1	0	9	3	20.0
ART duration (months)^6^										
**Median (IQR)**	36	5–61	34	14–49	58	35–89	51	27–75	44	16–76
**ART naïve or <3 months**	15	19.7	8	18.2	1	3.6	0	0	2	13.3
**≥3 months**	51	67.1	30	68.2	25	89.3	2	100.0	10	66.7
**Unknown**	10	13.2	6	13.6	2	6.9	0	0	3	20.0
Viral load (copies/ml)^6^										
**<150**	39	51.3	29	65.9	18	64.3	1	50.0	8	53.3
**150–999**	5	6.6	1	2.3	1	3.6	1	50.0	1	6.7
**≥1000**	23	30.3	8	18.2	1	3.6	0	0	4	26.7
**Unknown**	9	11.8	6	13.6	8	28.6	0	0	2	13.3

Abbreviations

Tuberculosis (TB), latent TB infection (LTBI), interquartile range (IQR), antiretroviral therapy (ART), millilitre (ml).

1. Study participants without past or current active TB and with negative QuantiFERON TB GOLD PLUS reactivity.

2. Study participants with positive QuantiFERON TB GOLD PLUS reactivity, without past or current active TB.

3. Study participants reporting previous treatment for active TB.

4. Study participants diagnosed with active during the pregnancy or within three months of delivery.

5. Excluded because of lack of study visit after delivery. In this group, 244 (65.8%) were classified as TB-uninfected, 117 (31.5)% as having LTBI, 9 (2.4%) reported previous active TB and 1 (0.3%) were diagnosed with active TB in connection to pregnancy, respectively.

6. Concerning HIV-positive participants only.

At inclusion, median age was 25 years (interquartile range [IQR] 22–28), with median gestational age 18 weeks (IQR 14–21) (Table 1). Of 150 (10.3%) HIV-positive participants, 17 (11.3%) were diagnosed with HIV in connection to the current pregnancy, 108 (72.0%) were on antiretroviral therapy for ≥3 months, and 87 (58.0%) had plasma HIV RNA levels <150 copies/mL. Five-hundred-five participants (34.5%) were primigravidae. The median gestational age at delivery was 40 weeks (IQR 38–42).

Four (0.3%) women were diagnosed with active TB during (3 pulmonary TB [2 HIV-positive, 1 HIV-negative], 1 TB pleuritis [HIV-negative]), and 68 (4.6%) had received treatment for active TB before pregnancy. LTBI was detected in 470 (32.1%) participants, and 921 (63.0%) were classified as TB-uninfected. A greater proportion of participants with LTBI were >25 years of age compared to TB-uninfected participants (52.8% *vs* 36.7%, p<0.001), and reported previous pregnancies more frequently (71.5% *vs* 61.3%, p<0.001) (Table 1).

No maternal deaths were observed among the included women. In total, 64 (4.4%) pregnancies resulted in intrauterine demise, among which 11 (17.2%) were classified as spontaneous abortions and 47 (73.4%) as stillbirths, and 6 deaths (9.4%) occurred an at unknown gestational age (Table 2). Fifteen neonatal deaths were observed (1.0%). One-hundred-sixty-seven (11.4%) and 22 (1.5%) women delivered by caesarean section and instrumental delivery, respectively, and 110 (7.5%) were hospitalized for ≥24 hours in connection to delivery. Three participants reported foetal congenital malformation, these pregnancies resulted in stillbirth.

**Table 2:** Pregnancy outcomes with regard to TB infection category.

Characteristics	TB-uninfected^1^ N = 921		LTBI^2^ N = 470		Previous active TB^3^ N = 68		Active TB diagnosed during pregnancy^4^ (n = 4)	
**Offspring vital outcome**	N	%	N	%	N	%		
**Spontaneous abortion^5^**	5	0.5	5	1.1	1	1.5	0	0
**Stillbirth^6^**	25	2.7	19	4.0	3	4.4	0	0
**Fetal demise, unknown gestational age**	4	0.4	2	0.4	0	0	0	0
**Neonatal death ≤28 days ^7^**	10	1.1	5	1.1	0	0	0	0
**Neonatal death ≤7 days**	7	0.7	0	0	0	0	0	0
**Survival >28 days**	877	95.2	439	93.4	64	94.1	4	100.0
**Delivery method**								
**Spontaneous vaginal**	772	83.8	388	82.6	55	80.9	4	100.0
**Instrumental**	10	1.1	8	1.7	4	5.9	0	0
**Emergency CS**	74	8.0	40	8.5	3	4.4	0	0
**Elective CS**	32	3.5	16	3.4	1	1.5	0	0
**Unknown**	33	3.6	18	3.8	5	7.4	0	0
**Hospitalization >24 hours**								
**Yes**	71	7.7	36	7.7	2	2.9	1	25.0
**No**	822	89.3	414	88.1	63	92.6	3	75.0
**Unknown**	28	3	19	4.3	3	4.4	0	0

Abbreviations: Tuberculosis (TB), latent TB infection (LTBI), caesarean section (CS).

1. Study participants without past or current active TB and with negative QuantiFERON TB GOLD PLUS reactivity.

2. Study participants with positive QuantiFERON TB GOLD PLUS reactivity, without past or current active TB.

3. Study participants reporting previous treatment for active TB.

4. Study participants diagnosed with active during the pregnancy or within three months of delivery.

5. Fetal demise occurring <20 weeks of gestation.

6. Fetal demise occurring from 20 weeks of gestation until and including the day of delivery.

7. Neonatal death before or at 28 days after delivery, not including the day of delivery.

### Stillbirth and neonatal death with regard to maternal LTBI

Rates of spontaneous abortion (5/470, 1.1% vs 5/921, 0.5%) and stillbirth (19/470, 4.0% vs 25/921 (2.7%) were slightly higher in participants with LTBI compared to TB-uninfected participants. Stillbirth was not significantly associated with maternal LTBI in simple (OR 1.51, p = 0.18) or multivariable logistic regression analysis adjusting for HIV status, age and gravidity (AOR 1.38, 95% CI 0.73–2.57, p = 0.30) (Table 3). In sensitivity analysis with additional adjustment for maternal education and marital status, number of rooms and presence of electricity and latrine in the household, similar results were obtained (AOR 1.40, 95% CI 0.73–2.63, p = 0.30, S1 Table).

**Table 3:** Crude and adjusted odds ratios for stillbirth (≥20 weeks of gestation) with respect to maternal TB infection category, maternal age, gravidity and HIV-status.

Characteristic	OR	95% CI	p	AOR	95% CI	p
**TB category**						
**TB uninfected^1^**	Ref	Ref		Ref	Ref	
**LTBI^2^**	1.51	0.81–2.76	0.18	1.38	0.73–2.57	0.30
**Previous active TB^3^**	1.65	0.38–4.85	0.43	1.47	0.33–4.68	0.56
**Current active TB^4^**	NA	NA	NA	NA	NA	NA
**Age (years)**						
**<21**	Ref	Ref		Ref	Ref	
**21–25**	0.56	0.22–1.47	0.22	0.70	0.27–1.85	0.45
**26–30**	1.04	0.46–2.59	0.92	1.55	0.62–4.14	0.36
**>30**	2.82	1.11–7.47	0.03	4.54	1.55–13.8	0.006
**First pregnancy**						
**Yes**	1.42	0.78–2.56	0.24	2.32	1.15–4.64	0.02
**No**	Ref	Ref		Ref	Ref	
**HIV status**						
**HIV negative**	Ref	Ref		Ref	Ref	
**HIV positive**	1.28	0.48–2.87	0.57	1.02	0.36–2.47	0.97

Adjustments were made for maternal age, gravidity and HIV status. Due to missing data on covariates, 11 cases (0.8%) were excluded from multivariable analysis.

Abbreviations: Odds ratio (OR), confidence interval (CI), adjusted odds ratio (AOR), Tuberculosis (TB), latent TB infection (LTBI), caesarean section (CS).

1. Study participants without past or current active TB and with negative QuantiFERON TB GOLD PLUS reactivity.

2. Study participants with positive QuantiFERON TB GOLD PLUS reactivity, without past or current active TB.

3. Study participants reporting previous treatment for active TB.

4. Study participants diagnosed with active during the pregnancy or within three months of delivery.

The proportions of neonatal death (1.1% *vs* 1.1%) were similar among women with LTBI compared to those without TB infection. Similar findings were obtained for rates of hospitalization (7.7% *vs* 7.7%) and caesarean section (11.9% *vs* 11.5%).

### Foetal and neonatal death in participants with past or current active TB

No foetal or neonatal deaths were reported among the four women with active TB during the pregnancy. Among participants with previous treatment for active TB, 3/68 (4.4%) pregnancies resulted in stillbirth (OR 1.65, AOR 1.47, 95% CI 0.33–4.68, p = 0.56, Table 3). Rates of hospitalization (2/68, 2.9%) and caesarean section (4/68, 5.9%) were slightly lower than in TB-uninfected participants. There were no neonatal deaths in this group.

## Discussion

In this cohort of Ethiopian pregnant women categorized for TB infection, rates of foetal death were slightly higher among women with LTBI as compared to those without TB infection, but this difference did not reach statistical significance.

The risk of reproductive morbidity and mortality is unevenly distributed globally, with 98% of stillbirths occurring in low- and middle-income countries [31]. Poverty and limited access to healthcare contribute to this phenomenon. However, women of African origin immigrating to high-income countries such as Sweden have an increased risk of stillbirth compared to native Swedes [32]. Several factors, including socio-economic conditions, quality of care provided and health-seeking behaviour, may be involved in this phenomenon. In addition, infectious diseases have been proposed to contribute to an excess risk of adverse pregnancy outcome in migrants in high-income countries [33]. In this context, TB infection is of interest, since it is common in sub-Saharan Africa, and since active TB is a recognized risk factor for adverse pregnancy outcomes [9].

Pregnant women have increased susceptibility to various infectious diseases, which may in turn contribute to pregnancy complications, with variable pathogenetic mechanisms involving placental or foetal invasion as well as systemic inflammatory responses disturbing placental development [7,34–40]. While placental or foetal involvement of TB has been rarely described [41], the mechanisms leading to the majority of TB-associated pregnancy complications are not fully explained.

Interestingly, elevated levels of biomarkers of systemic inflammation have also been reported in persons with LTBI [42–45], and a recent study revealed differences in peripheral blood cytokine profiles among Indian pregnant women with LTBI compared to TB-uninfected women [46]. Furthermore, recent observational studies have suggested associations between LTBI and myocardial infarction and atherosclerosis [47,48], which has been suggested to be linked to chronic low-grade inflammation. These observations led us to hypothesize that low-grade systemic inflammation in people with LTBI might contribute to pregnancy-related disorders as well.

Although we did not find a significant difference in rates of stillbirth with regard to LTBI status in our study population, it is possible that a larger sample size could have revealed such an association. In addition, associations between LTBI and certain types of pregnancy complications may have been missed in the current study, due to restricted sample size and insufficient information to determine specific pregnancy complications and causes of stillbirth among participants.

Our study participants were recruited at public ANC clinics in a TB-endemic urban uptake area in Ethiopia [23]. All participants were systematically screened for LTBI using a modern interferon-γ release assay. Apart from the limitations mentioned above, nearly 20% of the women did not have a physical post-partum study visit. Although adverse pregnancy outcomes may have been more common in this group, loss to follow-up was distributed similarly with respect to LTBI status and other characteristics; therefore, we consider it unlikely that this led to bias regarding the association between foetal death and LTBI. The total proportion of stillbirths occurring after 28 gestational weeks (including intrapartum deaths) was comparable to estimations for sub-Saharan Africa [31].

### Conclusions

In this cohort of pregnant Ethiopian women, LTBI was not significantly associated with stillbirth nor neonatal death. Further studies with larger sample size and ascertainment of other types of pregnancy complications are required to determine whether LTBI is involved in stillbirth or other specific types of adverse pregnancy outcomes.

## Supporting information

S1 Checklist (DOCX)

S1 Table Odds ratios for foetal death with respect to maternal TB infection category with broader adjustment. Abbreviations: OR: Odds Ratio CI: Confidence Interval LTBI: Latent Tuberculosis Infection.
(DOCX)

S1 File Raw data. (XLSX)
