# Supplementary material for: Tuberculosis infection and stillbirth in Ethiopia—A prospective cohort study
Source: PLoS One. 2022 Apr 11;17(4):e0261972. doi: 10.1371/journal.pone.0261972 (PMC9000061; doi:10.1371/journal.pone.0261972)
Supplement: S1 Table — Abbreviations: OR: Odds Ratio CI: Confidence Interval LTBI: Latent Tuberculosis Infection. (DOCX) [file pone.0261972.s002.docx]

### S1 Table. Odds ratios for stillbirth (≥20 weeks of gestation) with respect to maternal TB infection category with broader adjustment.

| Characteristic | AOR | 95% CI | | p |
| --- | --- | --- | --- | --- |
| TB category |  |  |  |  |
| TB uninfected^1^ | Ref | Ref | Ref | Ref |
| LTBI^2^ | 1.40 | 0.73 | 2.63 | 0.30 |
| Previous active TB^3^ | 2.09 | 0.45 | 7.12 | 0.28 |
| Current active TB^4^ | NA | NA | NA | NA |
| Age (years) |  |  |  |  |
| <21 | Ref | Ref | Ref | Ref |
| 21-25 | 0.79 | 0.31 | 2.12 | 0.63 |
| 26-30 | 1.66 | 0.63 | 4.59 | 0.31 |
| >30 | 5.43 | 1.76 | 17.4 | 0.004 |
| First pregnancy |  |  |  |  |
| Yes | 2.82 | 1.35 | 5.91 | 0.006 |
| No | Ref | Ref | Ref | Ref |
| HIV status |  |  |  |  |
| HIV negative | Ref | Ref | Ref | Ref |
| HIV positive | 0.92 | 0.31 | 2.35 | 0.87 |
| Marital status |  |  |  |  |
| Single | NA | NA | NA | NA |
| Married | Ref | Ref | Ref | Ref |
| Divorced | 2.67 | 0.12 | 20.0 | 0.41 |
| Widow | NA | NA | NA | NA |
| Education |  |  |  |  |
| Illiterate | 0.80 | 0.26 | 2.02 | 0.66 |
| < 6 grades | 1.22 | 0.57 | 2.45 | 0.60 |
| 6-12 grades | Ref | Ref | Ref | Ref |
| Higher education | 0.14 | 0.007 | 0.65 | 0.05 |
| Rooms in residence |  |  |  |  |
| 1 | Ref | Ref | Ref | Ref |
| ≥2 | 1.31 | 0.68 | 2.54 | 0.43 |
| Electricity in residence |  |  |  |  |
| No | Ref | Ref | Ref | Ref |
| Yes | 1.84 | 0.37 | 3.36 | 0.56 |
| Latrine in residence |  |  |  |  |
| No | Ref | Ref | Ref | Ref |
| Yes | NA | NA | NA | NA |

Adjustments were made for maternal age, gravidity, HIV status, marital status, level of education, and number of rooms, access to electricity and latrine in the residence. Due to missing data on covariates, 32 cases (2.2%) were excluded from multivariable analysis.

Abbreviations: confidence interval (CI), adjusted odds ratio (AOR), Tuberculosis (TB), latent TB infection (LTBI)

1. Study participants without past or current active TB and with negative QuantiFERON TB GOLD PLUS reactivity.
2. Study participants with positive QuantiFERON TB GOLD PLUS reactivity, without past or current active TB.
3. Study participants reporting previous treatment for active TB.
4. Study participants diagnosed with active during the pregnancy or within three months of delivery.
